# A Comparative Study of the Influence of Various Fungal-Based Pretreatments of Grape Pomace on Phenolic Compounds Recovery

**DOI:** 10.3390/foods11111665

**Published:** 2022-06-06

**Authors:** Gordana Šelo, Mirela Planinić, Marina Tišma, Josipa Grgić, Gabriela Perković, Daliborka Koceva Komlenić, Ana Bucić-Kojić

**Affiliations:** Faculty of Food Technology Osijek, Josip Juraj Strossmayer University of Osijek, Franje Kuhača 18, 31000 Osijek, Croatia; gselo@ptfos.hr (G.Š.); mplanini@ptfos.hr (M.P.); mtisma@ptfos.hr (M.T.); jgrgic2@ptfos.hr (J.G.); gperkovic@ptfos.hr (G.P.); dkoceva@ptfos.hr (D.K.K.)

**Keywords:** grape pomace, solid-state fermentation, phenolic compounds recovery

## Abstract

Wineries produce considerable amounts of grape pomace, which is a readily available natural source of bioactive phenolic compounds. In this study, grape pomace was used as a substrate for the cultivation of eleven filamentous fungi (*Trametes versicolor* TV6, *Trametes versicolor* TV8, *Trametes versicolor* AG613, *Trametes gibbosa*, *Phanerochaete chrysosporium*, *Ceriporiopsis subvermispora*, *Pleurotus eryngii*, *Ganoderma lucidum*, *Ganoderma resinaceum*, *Humicola grisea*, and *Rhizopus oryzae*) under solid-state conditions (SSF) for 15 days with the aim of improving the recovery of the individual phenolic compounds. Twenty-one phenolic compounds were quantified and the recovery of seventeen of them (gallic acid, ellagic acid, *p*-hydroxybenzoic acid, syringic acid, vanillic acid, 3,4-dihydroxybenzoic acid, ferulic acid, *o*-coumaric acid, *p*-coumaric acid, epicatechin gallate, galocatechin gallate, quercetin, kaempferol, procyanidin B1, procyanidin B2, resveratrol, and ε-viniferin) were positively affected by SSF. Ellagic acid is the most recovered compound, whose content increased 8.8-fold after 15 days of biological treatment with *Ceriporiopsis subvermispora* compared to the untreated initial sample. Among the microorganisms tested, the fungi *Pleurotus eryngii* and *Rhizopus oryzae* proved to be the most effective in increasing the recovery of most phenolic compounds (1.1–4.5-fold). In addition, the nutrient composition (proteins, ash, fats) of grape pomace was positively affected by the biological treatments.

## 1. Introduction

After processing grapes into wine, about 20–30% remains as grape pomace (GP), which contains valuable ingredients in its chemical composition (fibers, proteins, lipids, natural antioxidants in the form of phenolic compounds, etc.), but is still underutilized [1,2]. GP is usually improperly disposed of in the environment or burned without a clear waste management strategy, which can have numerous negative impacts on the environment (impending the germination of plants in the soil, occurrence of undesirable odors) and economic losses [3]. It is still widely used in distilleries for alcohol production, while a smaller part is used for composting or dried and used as fertilizer or animal feed [4,5]. In line with sustainable development and increasing consumer demand for the use of natural rather than synthetic resources, there is great interest in the reuse of GP for various purposes, such as the production of fertilizers, animal feed, functional foods, pharmaceuticals, cosmetics, industrial enzymes, composites, biopolymers, as biomass for biofuel production, to its use for mushroom cultivation, and as an additive to improve building insulation [1,6,7,8]. These alternative uses of GP, as well as its market potential, are still under development, but some products are already available on the market, such as Bioflavia, an organic red grape skin powder used as a dietary supplement, grape seed extract capsules (Ecovitis^TM^) rich in oligomeric proanthocyanidins, and grape seed oil [9,10].

It is known that GP contains a high content of natural bioactive compounds, the extraction of which has recently become an important research topic worldwide. These compounds are secondary plant metabolites which mainly include phenolic acids (hydroxybenzoic acids and hydroxycinnamic acids), flavonoids (catechins, flavonols, anthocyanins, and procyanidins), and stilbenes, which have beneficial effects on human health, such as prevention and treatment of diabetes and cardiovascular diseases, antibacterial, antitumor, anti-inflammatory and antioxidant effects, etc. [2,11,12]. About 70% of the phenolic compounds content present in grapes remains in the GP after its processing into wine [2].

However, complete recovery of bioactive compounds by conventional extraction methods can be difficult because these compounds are often entrapped in the lignin fraction and are present in an insoluble bound form of conjugates with sugars, fatty acids, or amino acids that are part of the plant cell wall [12,13]. One of the most abundant polymers that make up the plant cell wall is lignin, a complex amorphous heteropolymer composed of phenylpropanoid units. It binds hemicellulose to cellulose in the plant cell wall [14]. The lignin content in GP ranges from 11.6 to 41.3% [15,16]. In order to degrade the complex lignocellulosic structure and release the phenolic compounds from the lignin structure to make them more accessible for extraction, pretreatment of GP is required. Pretreatment of lignocellulosic materials is carried out by physical, chemical, biological methods, and by a combination of these methods [17]. Biological methods such as solid-state fermentation (SSF) provide an alternative to the recovery of natural phenolic compounds and are a useful technique and an environmentally friendly bioprocess that can yield high-value products with potential industrial applications [18]. Filamentous fungi are the most commonly used microorganisms for SSF, capable of synthesizing enzymes involved in the degradation of complex lignocellulosic material and the release of phenolic compounds. GP could be a good substrate for SSF to obtain phenolic compounds due to its chemical composition suitable for microbial growth [13]. Some studies have investigated the use of GP as a substrate in SSF with the aim of recovering phenolic compounds [12,13,19], but there is not yet much detailed information on the effects of the biological treatment of GP on the profile of phenolic compounds. To our knowledge, this is the first study to screen the profiles of individual phenolic compounds in extracts obtained from a biologically untreated sample of GP and from a 15-day treatment of GP by eleven filamentous fungi cultured under SSF conditions: *Trametes versicolor* TV6 (TV6), *Trametes versicolor* TV8 (TV8), *Trametes versicolor* AG613 (TV AG613), *Trametes gibbosa* (TG), *Phanerochaete chrysosporium* (PC), *Ceriporiopsis subvermispora* (CS), *Pleurotus eryngii* (PE), *Ganoderma lucidum* (GL), *Ganoderma resinaceum* (GR), *Humicola grisea* (HG), and *Rhizopus oryzae* (RO).

## 2. Materials and Methods

### 2.1. Standards and Reagents

The following standards and reagents were used in this study: analytical grade ethanol was acquired from Gram Mol Ltd. (Zagreb, Croatia), ultra-gradient grade methanol from J.T. Baker (Arnhem, The Netherlands), HPLC grade acetonitrile from Fisher Chemical (Loughborough, UK), and glacial acetic acid from Macron Fine Chemicals (Gliwice, Poland). Gallocatechin gallate, epicatechin gallate, (−)-epicatechin, (+)-catechin hydrate, caffeic acid, ellagic acid, gallic acid, resveratrol, syringic acid, kaempferol, *o*-coumaric acid, *p*-coumaric acid, ferulic acid, rutin hydrate, and *p*-hydroxybenzoic acid were purchased from Sigma Aldrich (Saint Louis, MO, USA). Procyanidin B1 and procyanidin B2 were obtained from Extrasynthese (Genay, France). Quercetin, 3,4-dihydroxybenzoic acid, and vanillic acid were obtained from Acros Organics (Geel, Belgium). ε-Viniferin was obtained from AppliChem (Darmstadt, Germany). Tyrosol, epigallocatechin, chlorogenic acid, sinapic acid, *p*-hydroxyphenylacetic acid, and myricetin were obtained from Sigma Aldrich (Saint Louis, MO, USA). Tween-80, DL-arabinose, maltotriose, L-rhamnose, sodium sulfite and n-octanol were obtained from Sigma Aldrich (Saint Louis, MO, USA). Cellulose, D(+)-xylose and iron(II)sulfate heptahydrate were obtained from Kemika (Zagreb, Croatia). D(−)-fructose, D(−)-ribose, D(+)-cellobiose, D(+)-galactose, D(+)-maltose monohydrate, D(+)-mannose, D(+)-sucrose and copper sulfate were obtained from Acros Organics (Geel, Belgium). D(+)-glucose was obtained from Gram Mol Ltd. (Zagreb, Croatia). Potassium hydrogen phthalate, Folin–Ciocalteu reagent and sodium carbonate anhydrous were acquired from Kemika (Zagreb, Croatia). Aluminum chloride hexahydrate was acquired from Alfa Aesar GmbH & Co KG (Kandel, Germany), and sodium nitrite, sodium sulfate, sodium hydroxide and acetone from Gram Mol Ltd. (Zagreb, Croatia). Hydrochloric acid, sulfuric acid, n-butanol and n-hexane were obtained from Carlo Erba Reagents GmbH (Emmendingen, Germany). Water was deionized in a Milli-Q water purification system (Millipore, Bedford, MA, USA) and HNO_3_ was purchased from Merck (Darmstadt, Germany).

### 2.2. GP Sample and Microorganisms

The sample GP is the residue from processing red grapes of the Cabernet Sauvignon variety into wine. GP was obtained from a local winery in eastern Croatia (Erdut vineyards, October harvest 2016) and consists of skin, pulp, seeds and stems. After collection, GP was stored at −20 °C before being used as substrate in SSF.

Biological treatment of GP was performed using eleven different filamentous fungi: TV6, TV8, TV AG613, TG, PC, CS, PE, GR, GL, HG, and RO. All microorganisms were cultured on potato dextrose agar (PDA) medium at 27 °C for 7 days, while RO was also cultured on PDA medium at 27 °C for 3 days.

### 2.3. Biological Pretreatment of GP by Eleven Filamentous Fungi

Next, 50 g of defrozen and coarsely crushed GP using a blender (Philips, HR 2860) was mixed with 30 mL of distilled water in 720 mL laboratory jars, autoclaved (121 °C/15 min), and allowed to cool overnight. GP was inoculated with 5 mycelial plugs of filamentous fungi (diameter 1 cm) suspended in 10 mL of sterile distilled water. In the case of RO, the spore suspension was prepared by resuspending the spores formed on PDA on Petri dishes in a Tween-80 solution (0.1% *v/v*). The moisture content of the substrate after inoculation was 65–75%. Experiments were performed separately for each fungus under the same conditions: incubation at 27 °C in an incubator with a fan set to 10% (KB 115, BINDER GmbH, Tuttlingen, Germany) for 1–5, 10 and 15 days. The height of the substrate layer in the jars was 4–4.5 cm. The control sample GP was prepared in the same way, except that the same amount of sterile water was added instead of the spore suspension, corresponding to day ‘0’. After each day of biological treatment, the jars containing the sample were again removed from the incubator and sterilized to complete the SSF process. The samples were then dried for 48 h at room temperature and ground to a particle size of ≤1 mm using an ultracentrifugal mill (Retsch ZM200, Haan, Germany). The dry matter content of the samples was in the range of 88–95%. The samples prepared in this way were stored at +4 °C until extraction and further analysis (chemical composition, profile of phenolic compounds).

### 2.4. Preparation of GP Extracts

Then, 1 g of dried and grounded GP (≤ 1 mm particle size) was extracted with 40 mL of ethanol: water (1:1) in stoppered flasks at 80 °C. Extractions were performed in a water bath (Julabo, SW-23, Seelbach, Germany) by shaking at 200 rpm for 120 min in three replicates. After extraction, samples were centrifuged at 10,000× *g* for 10 min (Z 326 K, Hermle Labortechnik GmbH, Wehingen, Germany). The supernatant was decanted and used for the determination of phenolic compounds content.

Extracts for measurement of individual sugar concentration were prepared by extraction of one gram of milled and dried GP with 25 mL of distilled water in sealed flasks. Extraction was performed in water bath by shaking at 170 rpm for 30 min, at 30 °C in three replicates. After extraction, the suspension was centrifuged at 10,000× *g* for 10 min (Z 326 K, Hermle Labortechnik GmbH, Wehingen, Germany). The supernatant was used for determination of sugar content.

### 2.5. Chemical Composition of GP

The chemical composition of the studied GP was expressed on a dry basis of the sample, and all measurements were performed in triplicate.

#### 2.5.1. Dry Matter Content

Dry matter content was determined by the thermogravimetric method using a fast moisture analyzer (HR-73, Mettler Toledo, Zürich, Switzerland). The sample was placed on an aluminum plate (about 3 g), which was placed directly on the integrated scale in the drying chamber, where drying to a constant mass was carried out. The drying conditions were: standard method, drying temperature 105 °C, and process termination criterion (switch-off 3: weight loss of 1 mg in 50 s).

#### 2.5.2. Ash Content

Ash content was determined gravimetrically by complete combustion of the samples (2 g) in a muffle furnace at 600 °C for 4 h according to method AACC-08-03 [20].

#### 2.5.3. Crude Proteins

Crude proteins were determined by the Kjeldahl method [21]. The sample was weighed on a cellophane sheet (0.2 g ± 0.1 mg), wrapped in cellophane, and placed in a glass tube. Then, 10 g of Na_2_SO_4_ and 0.1 g of CuSO_4_ were weighed in and also placed in the glass tube. Then, 15 mL of concentrated H_2_SO_4_ was added and the tubes were placed on a combustion rack and allowed to stand for 30 min. They were then gently shaken, connected to an exhaust system, and placed in a preheated block (420 °C) for combustion. Destruction took approximately one hour. After that, the samples were removed and cooled. Then, 75 mL of water was added to the test tube and distilled, collecting about 100 mL of distillate. An indicator was added to the distillate flask and titrated with 0.1 M NaOH, recording the volume of NaOH used for the titration. The percentage of nitrogen (N) and crude protein content (N × 6.25) were calculated.

#### 2.5.4. Free Fats Content

The free fat content was measured according to the Soxhlet standard method [22] using the Universal Extraction System (Büchi B-811 LSV, Flawil, Switzerland). Briefly, two grams of the sample were weighed into glass tubes, and extraction was performed with n-hexane for three hours. The free fats were collected in previously dried and weighed glass flasks.

#### 2.5.5. Total Organic Carbon (TOC) and Total Nitrogen (TN)

TOC and TN were analyzed by the 680 °C catalytic combustion oxidation method, and total carbon (TC) by catalytically aided combustion oxidation at 900 °C by a TOC analyzer (TOC-LCPH/CPN, SSM 5000A, Shimadzu, Japan).

#### 2.5.6. Neutral Detergent Fibers (NDF), Acid Detergent Fibers (ADF) and Acid Detergent Lignin (ADL)

NDF, ADF and ADL were determined according to the Van Soest method [23] using a fiber analyzer (FIWE 3, VELP Scientifica, Usmate Velate, Italy). A dried sample with a particle size ≤ 1 mm was weighed (1.0000 ± 0.0001 g) into pre-dried wells cooled in a desiccator (105 °C, 30 min), and placed on a VELP analyzer. For NDF analysis, 0.5 g Na_2_SO_3_, 100 mL NDF solution, and a few drops of n-octanol were added. The samples were heated to boiling and kept under reflux for 60 min. They were then filtered and washed with boiling water and cold acetone. For ADF analysis, 100 mL of the ADF solution and a few drops of n-octanol were added to the weighed samples, heated under reflux for 60 min, filtered, and washed with boiling water and cold acetone. Then they were dried at 105 °C for 8 h and weighed. To perform ADL analysis, ADF analysis was first performed and 25 mL of 72% H_2_SO_4_ was added to the flasks containing the filtered samples and washed with boiling water and cold acetone. Cold extraction was then performed for 3 h, stirring the samples every hour. After extraction, the samples were washed with boiling water until the acidic reaction was no longer present. Following the above protocols, the samples were dried at 105 °C for 8 h, cooled in a desiccator, and weighed. The NDF, ADF, and ADL were calculated and corrected for ash by burning the sample bottles in a muffle furnace at 550 °C for 2 h, cooling, and weighing in a desiccator. Hemicellulose content was calculated as the difference between NDF and ADF, cellulose content was calculated as the difference between ADF and ADL, while the ADL represents the content of lignin.

#### 2.5.7. Content of Individual Sugars

The individual sugar contents (sucrose, glucose, fructose, arabinose, xylose) were analysed by ultra-high performance liquid chromatography (UHPLC Nexera XR, Shimadzu, Japan) using a refractive index detector (RID) according to the method attached to the column. Briefly, sucrose content was determined using an Aminex^®^ HPX column (HPX-87H, 300 × 7.8 mm, Bio-Rad Laboratories, Hercules, CA, USA) with a mobile phase of 5 mM sulfuric acid and a flow rate of 0.6 mL/min at 40 °C for 60 min. The content of glucose, fructose, arabinose, and xylose was determined using a Nucleogel^®^ Sugar Pb column (VA, 300 × 7.8 mm, Macherey-Nagel GmbH & Co. KG, Dueren, Germany) with HPLC grade water as the mobile phase and a flow rate of 0.4 mL/min at 80 °C for 20 min. Data were recorded and analysed using the LabSolutions programme (version 5.87). Sugars in the extracts were identified by comparing the retention time and spectral data with an authentic standard.

#### 2.5.8. Content of Total Phenolic Compounds

Total phenolic compounds content (TPC) was determined by the Folin–Ciocalteu colorimetric method [24]. Briefly, 40 µL of extract was mixed with 3160 µL of water and 200 µL of Folin–Ciocalteu reagent. After 8 min, 600 µL of 20% (*w/v*) Na_2_CO_3_ was added and the mixture was incubated at 40 °C for 30 min. The absorbance was measured at 765 nm against a blank. Gallic acid was employed as a calibration standard.

#### 2.5.9. Content of Total Flavanoids

The content of total flavonoids (TF) was performed by the spectrophotometric method with the aluminum chloride [25]. Then, 500 µL of extract was mixed with 2000 µL of water. Next, 150 µL of 5% (*w/v*) sodium nitrite was added, and after 5 min, 150 µL of 10% (*w/v*) aluminium chloride as well. After exactly 6 min, 1000 µL of 1 M NaOH was added and the mixture was diluted with 1200 µL of distilled water. The absorbance was measured at 510 nm against a blank. (+)-catechin was used as a calibration standard.

#### 2.5.10. Content of Total Extractable Proanthocyanidins (TPA)

TPA were determined by a spectrophotometric method [26] based on the reaction with an acid-butanol solution and slightly modified. Briefly, 0.5 mL of GP extract was mixed with 5 mL of a ferrous sulphate solution prepared by dissolving FeSO_4_(H_2_O)_7_ in HCl-butanol solution and incubated at 95 °C for 15 min. After incubation, the reaction mixtures were cooled, and the absorbance was measured at 540 nm compared to a blank sample. The blank sample was prepared using distilled water instead of the extract.

### 2.6. Phenolic Content Analysis by UHPLC

Qualitative and quantitative analysis of phenolic compounds in GP liquid extracts was performed by UHPLC using a photodiode array (PDA) detector, according to the previously published method [27]. PDA detection was performed by scanning between 252 and 370 nm. Separation was performed using a reversed-phase Kinetex^®^ C18 core-shell column (100 × 4.6 mm, 2.6 µm, Phenomenex, Torrance, CA, USA). Samples were filtered through 0.45-µm membranes (Chromafil Xtra PTFE, Macherey-Nagel GmbH & Co. KG, Dueren, Germany). Separation was performed with a linear gradient of two solvents: Solvent A (1.0% acetic acid in water, *v/v*) and Solvent B (50% methanol, 50% acetonitrile, *v/v*). A linear gradient was performed at 30 °C with a flow rate of 1 mL/min from 5% to 30% B in 25 min, from 30% to 40% B in 10 min, from 40% to 48% B in 5 min, from 48% to 70% B in 10 min, from 70% to 100% B in 5 min, isocratic at 100% B for 5 min, followed by a return to baseline conditions (10 min) and column equilibration (12 min). The injection volume was 20 µL. Data were processed using LabSolutions 5.87 software. The phenolic compounds were identified by comparing their retention times and UV-Vis spectra with those of authentic standards analysed under the same chromatographic conditions. Quantification was performed according to the method of the external standard. Hydroxybenzoic acids were determined at 252–280 nm, hydroxycinnamic acids at 276–277 nm, flavan-3-ols at 273–277 nm, flavonols at 365–370 nm, procyanidins at 278 nm, and stilbenes at 305–323 nm. All experiments were performed in triplicate, and results were expressed as the mean of the replicate ± standard deviation (SD).

### 2.7. Statistical Analyses

Student’s *t*-test at 95% significance level (*p* < 0.05) using TIBCO Statistica software (TIBCO Software Inc., Palo Alto, CA, USA) was used to compare the mean values of individual phenolic contents between the zero day (untreated sample) and the day of fermentation when the maximum yield of phenolic compounds was obtained.

## 3. Results and Discussion

### 3.1. Chemical Composition of the GP

The chemical composition of GP depends on edaphoclimatic conditions, grape variety (white or red), type of tissue used (bark, seeds, pulp, stems), vinification conditions, and ripening stage [2]. The chemical composition of the biomass can strongly influence the whole SSF process, as it depends on the content of polysaccharides and other components that influence the growth of the microorganisms used and, consequently, the secretion of enzymes during SSF. Table 1 shows an example of the chemical composition of the raw material GP, used as substrate in SSF processes (day ‘0’) and the chemical composition of GP after 5, 10 and 15 days of SSF with TV6.

GP has a high lignin content, which makes it a matrix that is difficult to degrade. The lignin, cellulose, and hemicellulose content was calculated using NDF, ADF, and ADL, as indicated in Section 2.5.6. In this work, an increase in cellulose (from 13.88% to 17.98%) and lignin (from 32.36% to 44.71%) content was observed in GP after fungal treatment. Depending on the substrate, the proportion and composition of lignin may vary, resulting in different effects of fungal treatment. In addition, each fungal species has a specific strategy and shows different selectivity in lignin degradation [28]. Filippi et al. [29] conducted their studies on a GP with a lignin content of 34.79%, and Teles et al. [12] on a GP with a lignin content of 40.24%. However, the lignin content in GP can range from 11.6% to 41.3% depending on the grape variety [18]. To better understand the process of biological treatment and to see which components are actually degraded, it is important to present the data as absolute values using dry matter data. Van Kuijk et al. [28] gave the example of cellulose degradation and said that its absolute content cannot be increased during fungal treatment, but the values obtained show an increase. The same is for lignin content, which increased after 2 weeks of miscanthus fungal treatment. The reason for the increase in lignin and cellulose content could be the degradation of other components of the substrate when the fungus first consumes a large part of the readily available components [28,30]. Characteristics that make lignocellulosic biomass recalcitrant are the crystalline structure of cellulose, the hemicellulose that occurs between the macro- and microfibrils of cellulose, and the lignin that is present in the cellulose and hemicellulose matrix and is responsible for structural stability. The presence of lignin makes it difficult to access cellulose and hemicellulose. Therefore, the lignocellulosic biomass must be pretreated so that it can be further used to obtain the desired products, e.g., by converting cellulose and hemicellulose to simple sugars and fermenting them to produce biofuels, or to produce other high-value products, including bioactive phenolic compounds [12,31]. Table 1 shows that hemicellulose content decreased during SSF, which could be due to the action of hydrolytic enzymes such as xylanase produced by the filamentous fungi used. Xylanase acts on the degradation of xylan, the main unit of hemicellulose, as evidenced by the increase in the concentration of arabinose and xylose released from the hemicellulose structure. However, it should be noted that the microorganisms simultaneously use simple sugars as an energy source, which is a possible reason for the decrease in the concentration of certain single sugars during SSF [32,33]. Of the total thirteen sugar standards present in our collection, five sugars were quantified in the GP extracts (sucrose, glucose, fructose, arabinose, xylose). SSF can improve the nutrient composition of lignocellulosic biomass, as shown in this work, where a 40% increase in ash content and a 12% increase in crude protein content was obtained after 15 days of SSF.

A 20% increase in fat content was also noted. These data are consistent with the study on SSF of rice bran with RO, in which ash, crude protein, and fat content increased by 18.6%, 58.5%, and 124.5%, respectively [34], and with the work published by Altop et al. [35], which described an increase in the listed components after SSF of grape seeds with *Aspergillus niger*. The increase in crude protein content is probably due to mycelial development and/or enzyme production during filamentous fungal growth [35]. The increase in ash and lipid content could be related to fungal metabolism and hyphal and mycelial growth, since the cell wall of fungi contains both lipids and inorganic components. Inorganic anions and cations can form salts with basic and acidic components of the wall. Ash analysis of the cell wall of *Aspergillus flavus* detected elements such as K, Fe, Cl, Si, and Ca, while Mg, Cr, Cu, Al, Be, Mn, and Na, were also detected in *M. rouxi,* but some of them only in trace amounts [34,36]. SSF had no positive effect on the recovery of TPC, TF, and TPA, whose levels decreased by 33%, 27%, and 21%, respectively, of initial value after 15 days of SSF. The reason could be enzymes produced by the fermenting microorganism during its growth, and involved in the degradation of phenols, or stress-induced polymerization, which may cause the loss of free phenols during fermentation [37]. Meini et al. [38] also showed a decrease in TPC after SSF of GP with *Aspergillus niger* and *Aspergillus oryzae* at 68–81% substrate moisture content. At an initial substrate moisture content of 84%, fermentation with *A. oryzae* resulted in increased extractability of TPC compared to the untreated sample. The TOC results in Table 1 show the consumption of organic carbon by microorganisms during the SSF process, with a slight decrease in TOC after 15 days of fermentation. Moreover, carbon is constantly consumed during this process, but also released, for example, in the form of glucose, which can be seen from the increase in glucose concentration [39,40]. In the study published by Yang et al. [41], in which SSF of corn stalks was performed with *T. reesei*, a 39.66% decrease in TOC content was observed during the eighth day of fermentation. TN decreased after the first 5 days of fermentation and then increased until the 15th day of fermentation (Table 1), which is consistent with the crude protein results, and may be the reason for the increase in total nitrogen.

### 3.2. Weight Loss

Substrate weight loss is associated with the SSF process, in which filamentous fungi produce a complex system of enzymes that catalyze biotransformation processes and thus participate in the degradation of the solid substrate matrix. The degradation of cells or macromolecules of the substrate may release components bound to the matrix, such as antioxidants. Therefore, SSF can be a potential technology for upgrading such by-products, which can serve as a growth support and carbon source for microorganisms, thereby reducing the amount of by-products and obtaining high-value products such as bioactive compounds [42]. Biological treatment of GP over a 15-day period resulted in an average weight loss of GP to 28.8% when considering the final dry weight of the substrate relative to the initial dry weight of the substrate.

### 3.3. Phenolic Compound Profiles of GP before and after SSF

The phenolic profiles of GP extracts obtained from untreated and biologically treated GP samples were analyzed according to the UHPLC method described by Bucić-Kojić et al. [27]. Twenty-one phenolic compounds from twenty-seven available standards (in our collection) listed in Section 2.1. were detected and quantified in all analyzed extracts. Tyrosol, *p*-hydroxyphenylacetic acid, epigallocatechin, chlorogenic acid, sinapic acid, and myricetin were not detected in any of the samples analyzed. SSF had a positive effect on the recovery of 17 phenolic compounds (Table 2), whereas for four phenolic compounds (caffeic acid, catechin, epicatechin, rutin), no improvement in extraction yield was observed after SSF, therefore these data are not presented in this article.

**Figure 1:**
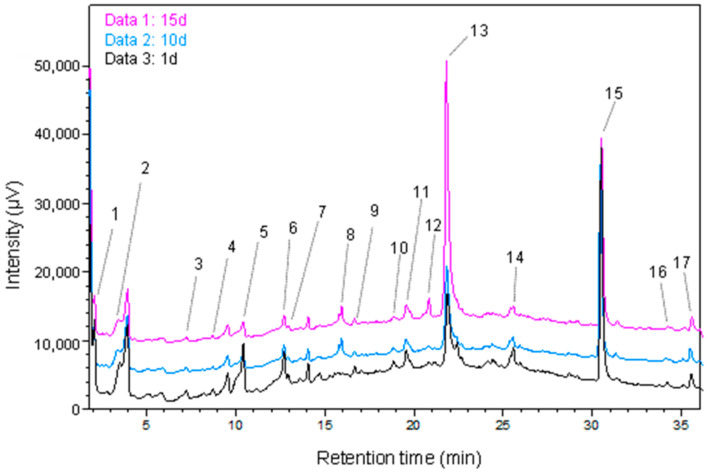
UHPLC chromatograms of phenolic compounds in GP extracts after 1, 10 and 15 days of SSF with CS: (1) gallic acid, (2) 3.4-dihydroxybenzoic acid, (3) *p*-hydroxybenzoic acid, (4) procyanidin B1, (5) vanillic acid, (6) syringic acid, (7) procyanidin B2, (8) *p*-coumaric acid, (9) gallocatechin gallate, (10) ferulic acid, (11) epicatechin gallate, (12) *o*-coumaric acid, (13) ellagic acid, (14) resveratrol, (15) quercetin, (16) ε-viniferin, (17) kaempferol.

Representative UHPLC chromatograms of the phenolic compounds are shown in Figure 1.

Table 2 shows the concentration range of each phenolic compound in untreated GP and their maximum concentration observed after SSF with a given filamentous fungus and duration of SSF. In general, the content of the 17 phenolic compounds listed in Table 2 increased from 1.12- to 8.76-fold after SSF, compared to the initial sample. Screening of the profile of phenolic compounds in the initial sample from GP and in the samples after biological treatment with 11 different filamentous fungi for 15 days showed that SSF had a positive effect on the recovery of phenolic compounds from GP. Therefore, among the 11 microorganisms used in SSF processes, PE and RO were found to be the most effective for the recovery of phenolic compounds from GP.

**Figure 2:**
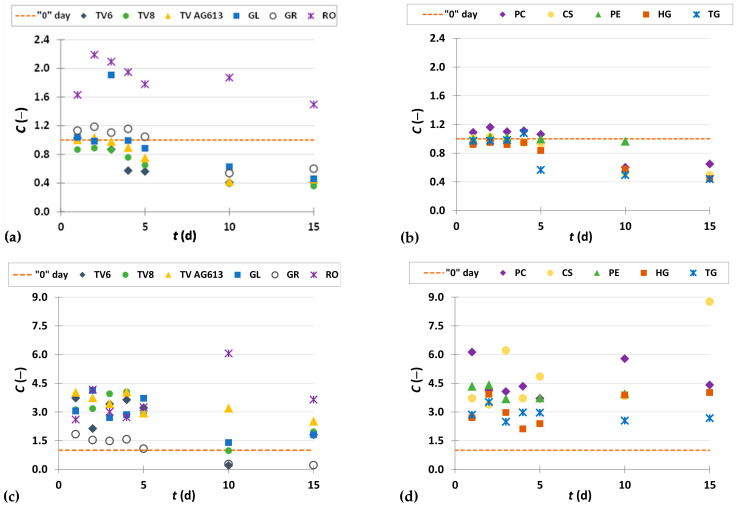
Dimensionless content (*C* = *C*_i_/*C*_o_) of gallic acid (**a**,**b**) and ellagic acid (**c**,**d**) in GP extracts before biological treatment (‘0’ day) and after 1–15 days of biological treatment with TV6, TV8, TV AG613, GL, GR, RO (**a**,**c**), and PC, CS, PE, HG, TG (**b**,**d**).

**Figure 3:**
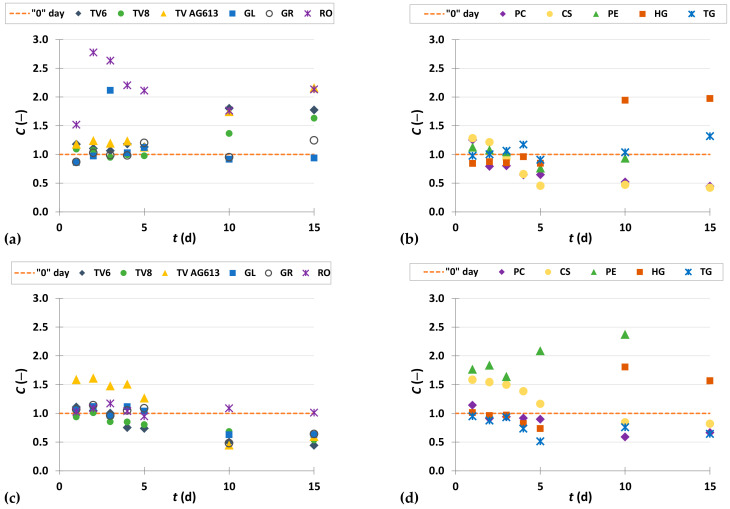
Dimensionless content (*C* = *C*_i_/*C*_o_) of *p*-hydroxybenzoic acid (**a**,**b**) and syringic acid (**c**,**d**) in GP extracts before biological treatment (‘0’ day) and after 1–15 days of biological treatment with TV6, TV8, TV AG613, GL, GR, RO (**a**,**c**), and PC, CS, PE, HG TG (**b**,**d**).

**Figure 4:**
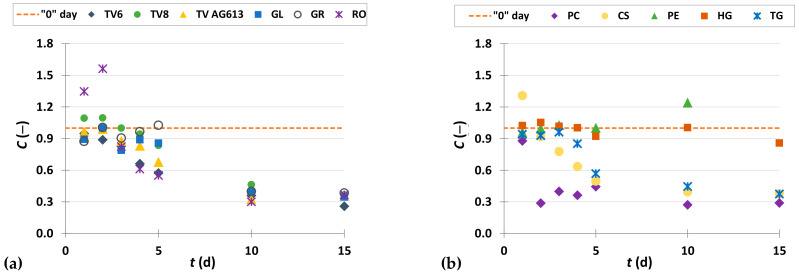

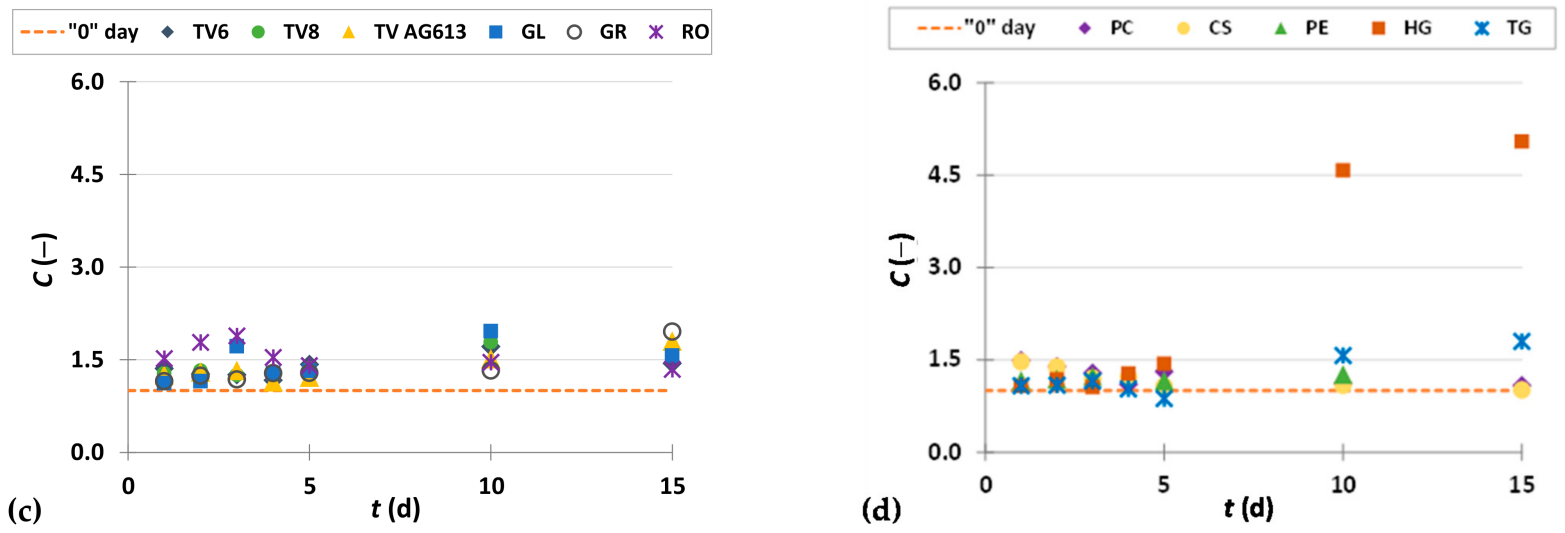
Dimensionless content (*C* = *C*_i_/*C*_o_) of vanillic acid (**a**,**b**) and 3,4-dihydroxybenzoic acid (**c**,**d**) in GP extracts before biological treatment (‘0’ day) and after 1–15 days of biological treatment with TV6, TV8, TV AG613, GL, GR, RO (**a**,**c**), and PC, CS, PE, HG, TG (**b**,**d**).

**Figure 5:**
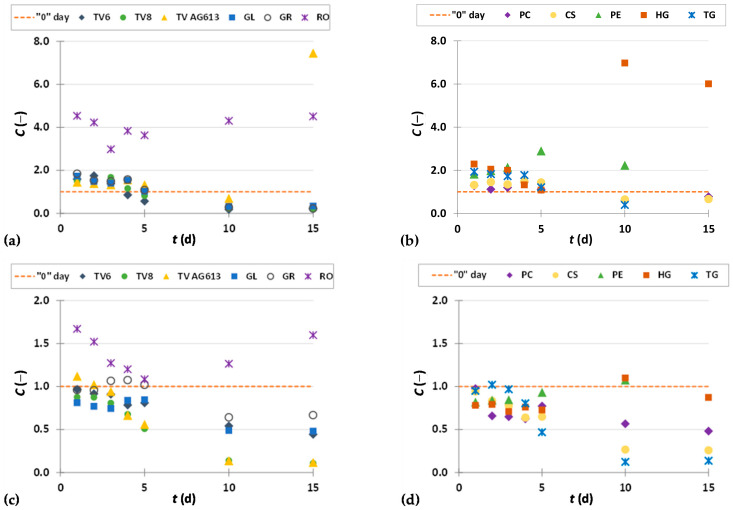
Dimensionless content (*C* = C_i_/C_o_) of *o*-coumaric acid (**a**,**b**) and *p*-coumaric acid (**c**,**d**) in GP extracts before biological treatment (‘0’ day) and after 1–15 days of biological treatment with TV6, TV8, TV AG613, GL, GR, RO (**a**,**c**), and PC, CS, PE, HG, TG (**b**,**d**).

**Figure 6:**
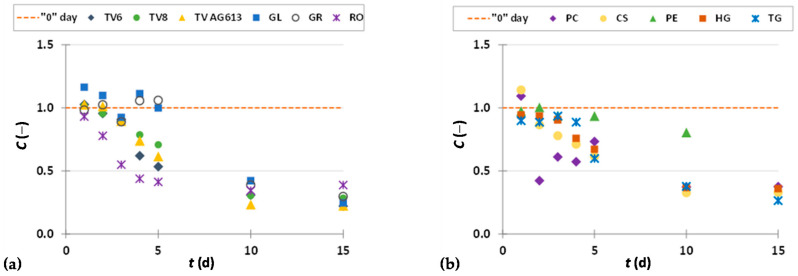
Dimensionless content (*C* = *C*_i_/*C*_o_) of ferulic acid in GP extracts before biological treatment (‘0’ day) and after 1–15 days of biological treatment with TV6, TV8, TV AG613, GL, GR, RO (**a**), and PC, CS, PE, HG, TG (**b**).

**Figure 7:**
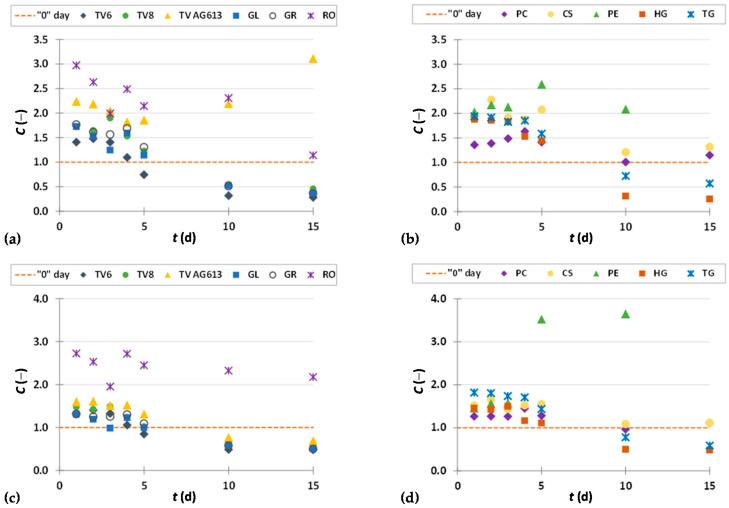
Dimensionless content (*C* = *C*_i_/*C*_o_) of epicatechin gallate (**a**,**b**) and gallocatechin gallate (**c**,**d**) in GP extracts before biological treatment (‘0’ day) and after 1–15 days of biological treatment with TV6, TV8, TV AG613, GL, GR, RO (**a**,**c**), and PC, CS, PE, HG, TG (**b**,**d**).

**Figure 8:**
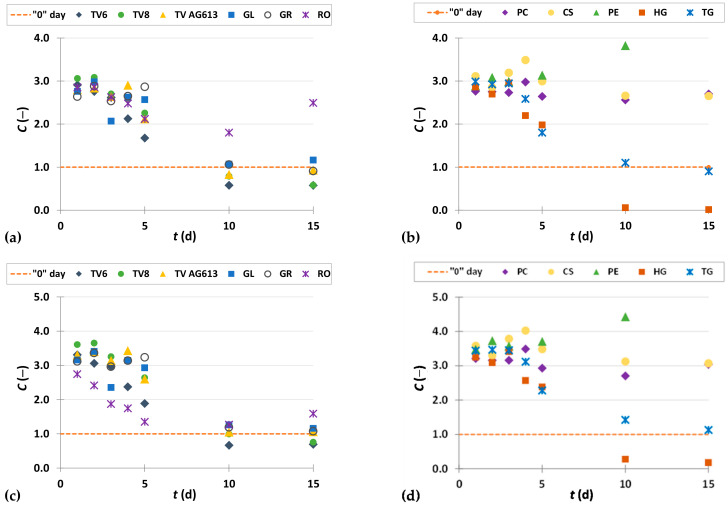
Dimensionless content (*C* = *C*_i_/*C*_o_) of quercetin (**a**,**b**) and kaempferol (**c**,**d**) in GP extracts before biological treatment (‘0’ day) and after 1–15 days of biological treatment with TV6, TV8, TV AG613, GL, GR, RO (**a**,**c**), and PC, CS, PE, HG, TG (**b**,**d**).

**Figure 9:**
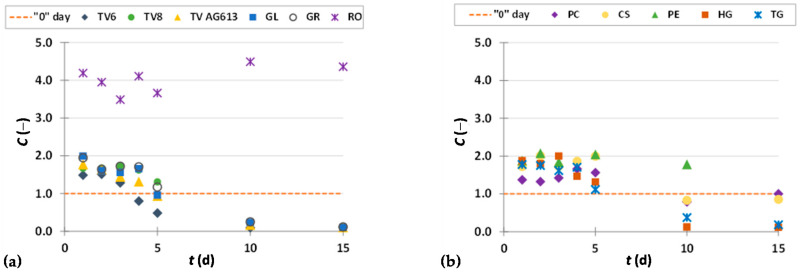

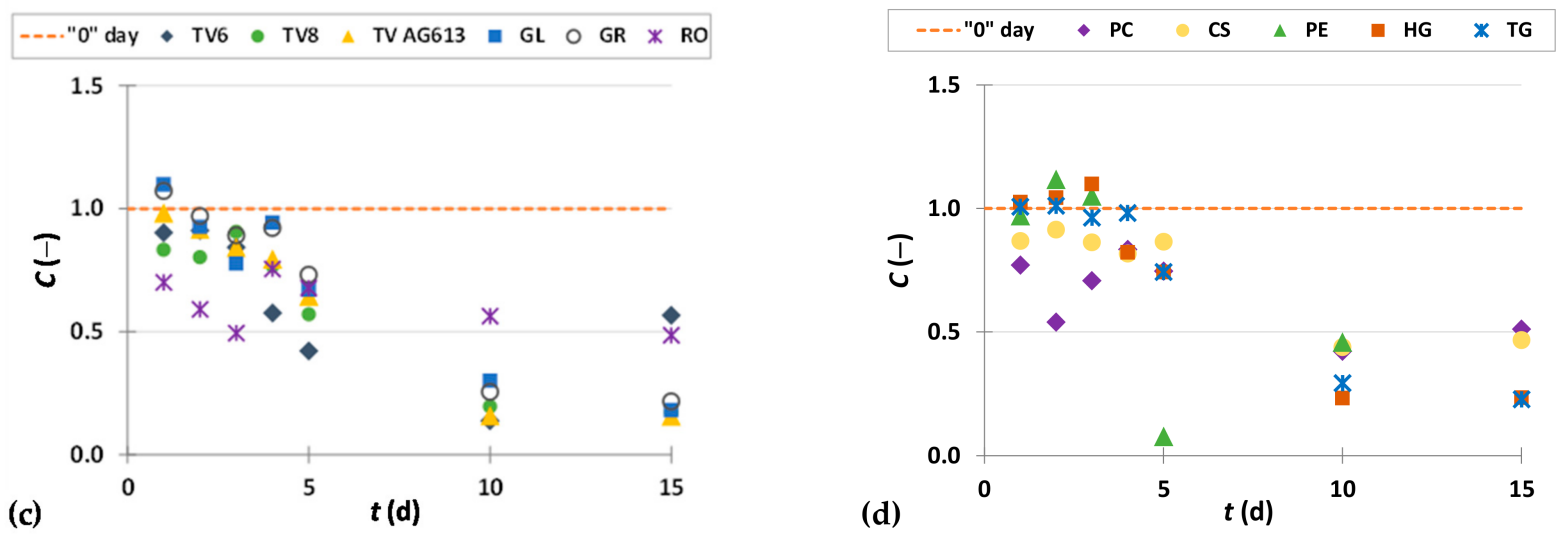
Dimensionless content (*C* = *C*_i_/*C*_o_) of procyanidin B1 (**a**,**b**) and procyanidin B2 (**c**,**d**) in GP extracts before biological treatment (‘0’ day) and after 1–15 days of biological treatment with TV6, TV8, TV AG613, GL, GR, RO (**a**,**c**), and PC, CS, PE, HG, TG (**b**,**d**).

**Figure 10:**
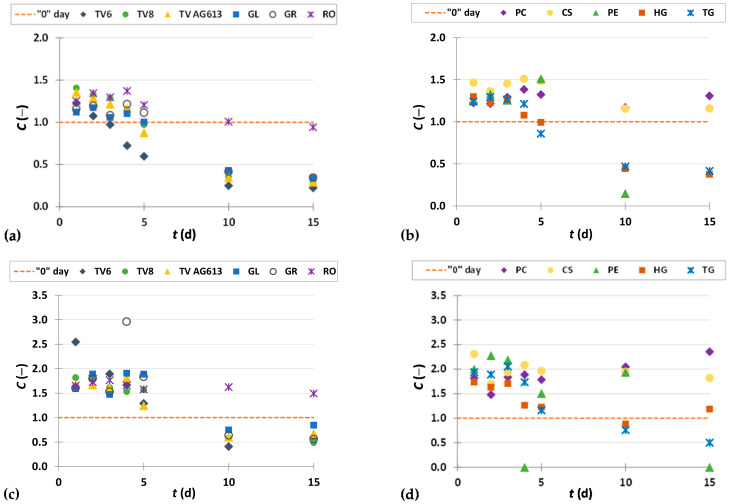
Dimensionless content (*C* = *C*_i_/*C*_o_) of resveratrol (**a**,**b**) and ε-viniferin (**c**,**d**) in GP extracts before biological treatment (‘0’ day) and after 1–15 days of biological treatment with TV6, TV8, TV AG613, GL, GR, RO (**a**,**c**), and PC, CS, PE, HG, TG (**b**,**d**).

To present the results more clearly and to better illustrate the effects of biological treatment by certain fungi on phenolic compound content in GP extracts, the results are presented as dimensionless content (*C* = *C*_i_/*C*_o_) of phenolic compounds (Figure 2, Figure 3, Figure 4, Figure 5, Figure 6, Figure 7, Figure 8, Figure 9 and Figure 10). The dimensionless content indicates the ratio between the phenolic compound content after biological treatment and the phenolic compound content in the untreated sample. The line in each graph shows the content of a particular phenolic compound in the untreated sample (‘0’ day). Values above the line indicate an increase, while values below the line indicate a decrease in the content of phenolic compounds after biological treatment.

#### 3.3.1. Phenolic Acids

Figure 2, Figure 3 and Figure 4 illustrate dimensionless content of hydroxybenzoic acids (gallic acid, ellagic acid, *p*-hydroxybenzoic acid, syringic acid, vanillic acid, 3,4-dihydroxybenzoic acid) in GP extracts before biological treatment (‘0’ day) and after 1–15 days of biological treatment with eleven filamentous fungi (TV6, TV8, TV AG613, GL, GR, RO, PC, CS, PE, HG, and TG). 

As shown in Figure 2a,b, biological treatment with RO, GL, GR, PC and TG had a positive effect on the recovery of gallic acid from GP during the first 5 days. The greatest increase in content of gallic acid by 2.2-fold was obtained after 2 days of biological treatment with RO. The content of gallic acid in the untreated sample was 267.77 ± 11.78 µg/g_db_, and in the sample after the third day of biological treatment, it was 586.43 ± 12.48 µg/g_db_ (Figure 2a, Table 2). Meini et al. [38] proved a significant increase in gallic acid when *A. niger* and *A. oryzae* were used for SSF of GP. Since GL produces the enzyme tannase, it is possible that its presence affects the release of gallic acid from tannic acid, hydrolyzable tannins, galloylated catechins and procyanidins present in GP [38,43]. Gallic acid has various biological functions in the treatment of numerous diseases such as cardiovascular disease, cancer, neurodegenerative disorders, and also an anti-ageing effect. It has antioxidant, anti-cancer, antibacterial, antifungal, antiviral, anti-inflammatory and anti-diabetic properties. Therefore, it is considered as a promising agent for the development of new drugs. Its large occurrence in nature and bioactivity have made it an important element in the development of new effective pharmacophores. Moreover, studies have shown that GA and its derivatives can selectively induce cancer cell death by apoptosis without harming healthy cells [44].

Ellagic acid is a dimeric derivative of gallic acid, which in plants is mainly ester-bound to sugars that are a constituent of ellagitannins (hydrolyzable tannins). It is widely used in food and biomedicine due to its beneficial effects on human health, but poor market availability and high prices limit its application [45]. Commercial ellagic acid is mainly obtained by extraction of ellagitannin-rich plant fractions using acid-methanol solvents and acid hydrolysis. However, due to problems in purification, low yields, and excessive costs, there is a need to develop alternative technologies that prove more efficient in achieving a cleaner product, higher yield, and lower cost on a larger scale. Therefore, intensive research is being conducted on biotechnological processes for its production [45]. In this study, biological treatment with all 11 microorganisms (Figure 2c,d) used resulted in an increase in ellagic acid content during all 15 days of fermentation, except for the 10th day of treatment with TV8 and TV6, when the ellagic acid content was lower than in the untreated sample. The most significant increase in ellagic acid content from 34.65 ± 3.66 µg/g_db_ to 303.72 ± 25.89 µg/g_db_ (8.8-fold) was obtained after 15 days of SSF with CS (Figure 2d,Table 2). 

*p*-Hydroxybenzoic acid is a phenolic acid found in fruits, vegetables and other plants. It is widely used in pharmaceutical, cosmetic and food industries (as antioxidant, preservative, fungicide in food, beverages, drugs and cosmetics). The antioxidant properties of *p*-hydroxybenzoic acid have a positive effect on human health, as it can eliminate free radicals associated with the physiology of various diseases, such as inflammatory and degenerative diseases [46,47]. Biological treatment of GP with RO, TV6, TV8, TV AG613, GL and GR resulted in an increase in the content of *p*-hydroxybenzoic acid after a certain duration of SSF, with the greatest increase (2.8-fold) in the content observed after three days of biological treatment with RO (Figure 3a). The content of *p*-hydroxybenzoic acid in the untreated sample was 5.05 ± 0.22 µg/g_db_, and in the sample after three days of biological treatment it was 14.37 ± 0.23 µg/g_db_ (Table 2). For filamentous fungi PC, PE and CS, significant increase was observed after 1 and 2 days of SSF, while for HG and TG, it was after 10 and 15 days of SSF (Figure 3b).

Syringic acid and its derivatives are found in various plant products and certain species of fungi. Together with numerous other phenolic compounds, syringic acid contributes to the structural integrity of lignin. Thanks to the phenolic core with various phenolic groups, syringic acid has a strong antioxidant capacity and anti-cancer, anti-inflammatory, anti-diabetic, neuroprotective, anti-endotoxic, hepatoprotective and cardioprotective properties [48]. It is precisely because of its therapeutic activities that it is of great importance to industry and its application in biomedicine. Due to its caries-reducing properties, it is used in the production of dental cement and is in great demand in the bioremediation industry, and photocatalytic ozonation and laccase activation in the pulp industry [48]. In this work, PE showed the best effect of all microorganisms (Figure 3c,d) on the recovery of syringic acid from GP, with a 2.4-fold increase in content after 10 days of biological treatment. In the untreated sample, the content of syringic acid was 86.37 ± 2.15 µg/g_db_, and in the sample after 10 days of biological treatment with PE, the content was 205.13 ± 0.01 µg/g_db_ (Table 2). Syringic acid and vanillic acid may affect the reduction of the quantitative marker of fibrosis, liver hydroxyproline, and suppress the aggregation of collagen [49].

Vanillic acid is an oxidized form of vanillin and a naturally occurring phenolic acid that has been shown to possess various pharmacological properties (antioxidant, antiapoptotic, hepatoprotective, cardioprotective, immunostimulant, anti-inflammatory, neuroprotective). It plays an important role in the prevention of inflammation and neurological diseases such as Alzheimer’s and Parkinson’s diseases [50]. As shown in Figure 4a,b, treatment of GP with RO, TV8, CS and HG resulted in an increase in vanillic acid content in the first two days of biological treatment, while treatment with PE resulted in an increase after 10 days. Cultivation of the remaining microorganisms on GP caused a decrease in vanillic acid content. The vanillic acid content in the untreated sample was 40.54 ± 0.21 µg/g_db_, and the greatest increase in content by 1.6-fold was observed after two days of biological treatment with RO (63.35 ± 1.57 µg/g_db_) (Figure 4a, Table 2). These results are in agreement with the data published by Bucić-Kojić et al. [51], in which an increase in vanillic acid extraction yield (up to 1.2-fold) was observed during the first 4 days of biological treatment of GP with TV6.

Protocatechuic acid (3,4-dihydroxybenzoic acid) not only possesses numerous pharmaceutical properties such as anticancer, antiviral, antibacterial, antidiabetic, nematicidal, antihyperlipidemic, and anti-atherosclerotic activities, but also can be used as a chemical building block in polymer and plastic production [52]. The content of 3,4-dihydroxybenzoic acid increased after biological treatment of GP with each microorganism during all 15 days of fermentation, except for the extract obtained after 5 days of biological treatment of GP with TG (Figure 4c,d). The highest increase (5.0-fold) in the extraction yield of 3,4-dihydroxybenzoic acid occurred after 15 days-treatment with HG (Figure 4d, Table 2).

Figure 5 and Figure 6 illustrate dimensionless content of hydroxycinnamic acids (*o*-coumaric acid, *p*-coumaric acid and ferulic acid) in GP extracts before biological treatment (‘0’ day) and after 1–15 days of biological treatment with eleven filamentous fungi (TV6, TV8, TV AG613, GL, GR, RO, PC, CS, PE, HG, and TG). Hydroxycinnamic acids form an important group of natural phenolic compounds, of which *p*-coumaric acid, caffeic acid, ferulic acid, and sinapic acid are the most abundant. Their antioxidant, anti-inflammatory and antimicrobial properties make them highly effective in preventing various diseases. They contribute to taste, color, nutritional value and health benefits, and play an important role in various biological processes in the human body [53].

Figure 5a,b show a significant increase in the extraction yield of *o*-coumaric acid due to biological treatment with RO, TV AG613, HG and PE, with the greatest increase recorded after 15 days of SSF with TV AG613 (7.5-fold) (Table 2). SSF of GP with RO had the best effect on increasing the extractability of *p*-coumaric acid, with its content increasing from 8.76 ± 0.70 µg/g_db_ to 14.63 ± 0.16 µg/g_db_ (1.7-fold) after the first day of fermentation (Figure 5c, Table 2). SSF with TV AG613 resulted in an increase in *p*-coumaric acid content after the first day with GR during the first 5 days (Figure 5c), and with HG and PE after 10 days (Figure 5d), whereas other filamentous fungi had no effect on increasing extractability from GP.

Ferulic acid is considered a powerful antioxidant because it has numerous physiological functions (anti-inflammatory, antimicrobial, anticancer, antiarrhythmic, antithrombotic, antidiabetic) [54]. It is mainly used in the cosmetic industry for its protective function for important skin structures such as elastin, collagen, keratinocytes, and fibroblasts, and is used in skin care formulations as a photoprotectant, to delay photoaging of the skin, and as a brightening component. It also accelerates wound healing, inhibits melanogenesis and promotes angiogenesis [49,54]. The increase of ferulic acid extractability was affected by biological treatment with GL and GR during the first 5 days, and by biological treatment of GP with PC and CS after the first day of fermentation, as shown in Figure 6a,b. Ferulic acid content decreased from the 5th to the 15th day of biological treatment. The greatest increase in ferulic acid yield was obtained after the first day of biological treatment with GL with an increase of 1.2-fold (Table 2).

#### 3.3.2. Flavan-3-ols

Flavan-3-ols represent the most reduced form of flavonoids and include a variety of monomeric catechins and oligomeric or polymeric procyanidins. These compounds play an important role in the quality and characteristics of wine and also have the function of a natural preservative. The flavan-3-ols of grape seeds possess various biological effects on human health and contribute to the health properties of wine [55]. Figure 7a–d show the dimensionless content of flavan-3-ols (epicatechin gallate, galocatechin gallate) in GP extracts before biological treatment (‘0’ day) and after 1–15 days of biological treatment with eleven filamentous fungi (TV6, TV8, TV AG613, GL, GR, RO, PC, CS, PE, HG and TG).

SSF had a positive effect on the recovery of epicatechin gallate during the first 5 days with all used microorganism, with the exception of day 5, when fermentation was performed with TV6 and the content of epicatechin gallate decreased compared to the untreated sample (Figure 7a,b). On the 10th and 15th day of biological treatment, the yield of epicatechin gallate extraction was increased by biological treatment with TV AG613, PE, CS and PC, while other filamentous fungi affected the decrease of epicatechin gallate content during the 10th and 15th day of treatment. The greatest increase in extraction yield was recorded in the SSF with TV AG613 after day 15, with a 3.1-fold increase in content (Figure 7a, Table 2). The recovery of gallocatechin gallate was improved by SSF with almost all microorganisms used during the first 5 days of fermentation (Figure 7c,d), and also on day 10 with PE when the greatest increase in extractability of this compound was obtained with a 3.7-fold increase compared to the untreated sample (Figure 7d, Table 2).

#### 3.3.3. Flavonols

Due to their prooxidant properties, flavonols may induce apoptosis and consenquently inhibit cancer development. They may participate in immune system function and protect living cells from free radicals. Properties such as antioxidant, neuroprotective, cardioprotective, anti-inflammatory, antibacterial, and antidiabetic are characteristic of kaempferol. Quercetin can increase reactive oxygen species and Ca^2+^ production. It also affects mitochondrial membrane potential and alters the expression of proteins related to apoptosis [56]. Figure 8a–d illustrate dimensionless content of flavonols (quercetin, kaempferol) in GP extracts before biological treatment (‘0’day) and after 1–15 days of biological treatment with eleven filamentous fungi (TV6, TV8, TV AG613, GL, GR, RO, PC, CS, PE, HG, and TG).

Biological treatment of GP with TV6, TV8, TV AG613, GL and GR during the first 5 days resulted in an increase in extractability of quercetin and kaempferol compared with the untreated sample. After 10 and 15 days of biological treatment with these microorganisms, the content of quercetin and kaempferol decreased (Figure 8a,c). Biological treatment of GP with PC, CS and PE resulted in increased extractability of quercetin (Figure 8b) and kaempferol (Figure 8d) during all 15 days of fermentation compared to the untreated sample. Their content increased during the first 5 days of biological treatment with HG and TG, and decreased after the 10th and 15th days (Figure 8b,d). The greatest increase in extractability of quercetin (3.8-fold) and kaempferol (4.4-fold) was obtained after 10 days of biological treatment with PE (Table 2).

#### 3.3.4. Procyanidins

Procyanidins are formed by oxidative condensation of (+)-catechin and/or (−)-epicatechin units. Consistently the most abundant procyanidin dimer in grape seeds is procyanidin B2 [55]. Procyanidins are characterized by antioxidant activity and the ability to specifically bind proteins and regulate cellular signaling pathways. Therefore, they are effective in preventing various diseases such as cancer, inflammation, cardiovascular disease, diabetes, and autoimmune diseases [57].

Figure 9a–d illustrate dimensionless content of procyanidins (procyanidin B1, procyanidin B2) in GP extracts before biological treatment (‘0’ day) and after 1–15 days of biological treatment with eleven filamentous fungi (TV6, TV8, TV AG613, GL, GR, RO, PC, CS, PE, HG, and TG).

Biological treatment with RO had the greatest influence on increasing the extractability of procyanidin B1 during all 15 days of SSF (Figure 9a). After ten days of fermentation, its content was 1368.26 ± 18.19 µg/g_db_, which is 4.5 times higher than the content in the untreated sample (304.27 ± 0.37 µg/g_db_).

In the case of procyanidin B2, biological treatment of GP with GL and GR after the first day of fermentation (Figure 9c) and treatment with PE and HG during the first three days of fermentation (Figure 9d) had a positive effect on increasing extractability. The greatest increase in the content of procyanidin B2 was obtained after the 2nd day of fermentation with PE (Figure 9d), when it increased 1.1-fold compared to the untreated sample (Table 2). In remaining days of fermentation, the content of procyanidin B2 decreased, as well as in the biological treatment with the other microorganisms during all 15 days of fermentation.

#### 3.3.5. Stilbenes

Stilbenes are derived from phenylpropanoid compounds, and grapes and red wine are some of the most important sources of stilbenes in the human diet. They have numerous biological activities such as antioxidant, neuroprotective, and antitumor effects, and are therefore beneficial for human health. In recent research, they are gaining great interest as potential anti-obesity agents. Among others, they may promote lipolysis, β-oxidation, thermogenesis, and mitochondrial biogenesis. Among the commonly identified stilbenes, resveratrol is the best-known compound [58]. Figure 10 illustrates dimensionless content of resveratrol (Figure 10a,b) and ε-viniferin (Figure 10c,d) in GP extracts before biological treatment (‘0’ day) and after 1–15 days of biological treatment with eleven filamentous fungi (TV6, TV8, TV AG613, GL, GR, RO, PC, CS, PE, HG, and TG).

Biological treatment of GP resulted in an increase in extractability of resveratrol, depending on the microorganism used and the day of fermentation (Figure 10a,b). Biological treatment with PE for 5 days had the greatest effect on increasing extractability when the content of resveratrol was 69.65 ± 0.97 µg/g_db_, which was 1.5-fold higher than the content of resveratrol in the untreated sample (46.07 ± 3.48 µg/g_db_) (Figure 10b, Table 2). The oxidative cyclization of resveratrol leads to its dimer, ε-viniferin, which exhibits potent activity against inflammatory and oxidative stress, and is considered a promising natural compound that could be used in the production of functional foods for the prevention of diseases caused by oxidative stress and inflammatory processes [59]. SSF of GP with GR, where the content increased from 17.52 ± 1.64 µg/g_db_ to 51.80 ± 0.35 µg/g_db_ after 4th day of fermentation, had the greatest effect on the increase of ε-viniferin content (Figure 10c, Table 2). During the first five days of SSF with all eleven filamentous fungi, the ε-viniferin content increased. An increase was observed on the 10th and 15th day of biological treatment with RO, PC, CS, PE and HG, while a decrease in the content was observed on the 10th and 15th day of SSF fermentation with TV8, GL, GR, TV6, TV AG613 and TG (Figure 10c,d).

Overall, SSF can be an effective technology for the recovery phenolic compounds, because during this process, microorganisms synthesize a complex system of enzymes involved in the degradation of the complex structure of heterogeneous substrates such as GP and lignocellulosic substrates in general [18]. This can lead to the release of phenolic compounds from the lignocellulosic structure, resulting in an increase in the content of phenolic compounds, usually due to hydrolytic enzymes [12], or degraded by enzymatic hydrolysis, leading to a decrease in the content of phenolic compounds [60]. However, in order to obtain the desired product, the process should be controlled, and it is important to stop it when the desired product is achieved [51]. Phenolic compounds possess strong biological activity, which is why their use is widespread in the food, cosmetic and pharmaceutical industries. They play a particularly important role in the prevention of various diseases. Gallic acid, for example, has antifungal, antimicrobial and antitumor properties, syringic acid has antioxidant, antimicrobial, anti-inflammatory and anti-endotoxic properties and is used for therapeutic purposes, while vanillic acid reduces collagen accumulation and hydroxyproline content [61]. The synergistic effect of the different subgroups of phenolic compounds and other bioactive compounds present in GP contributes to its better biological activity [62,63].

## 4. Conclusions

SSF is an environmentally friendly bioprocess capable of converting agricultural and food production residues into numerous high-value products, including phenolic com-pounds. Under experimental conditions, the SSF process improved the recovery of phenolic compounds when used as a pretreatment prior to extraction of grape pomace with 50% ethanol.

The greatest increase in phenolic content after SSF compared to the untreated initial sample was observed for ellagic acid (8.8-fold) when CS was used for 15 days. SSF with PE resulted in a significant increase in the content of six phenolic compounds, including syringic acid, gallocatechin gallate, quercetin, kaempferol, procyanidin B2, and resveratrol, by 2.4-, 3.7-, 3.8-, 4.4-, 1.1-, and 1.5-fold, respectively. SSF with RO resulted in a significant increase in the content of the following five phenolic compounds: gallic acid, *p*-hydroxybenzoic acid, vanillic acid, *p*-coumaric acid, and procyanidin B1 by 2.2-, 2.8-, 1.6-, 1.7-, and 4.5-fold, respectively. SSF with TV AG613 resulted in a significant increase in the content of *o*-coumaric acid by 7.5-fold and epicatechin gallate by 3.1-fold, while the content of 3,4-dihydroxybenzoic acid increased by 5-fold after 15 days of fermentation with HG. The extractability of ferulic acid improved by 1.2-fold, that of 3,4-dihydroxybenzoic acid by 5.0-fold, and that of ε-viniferin by 3.0-fold when GL, HG, and GR were used for SSF, respectively.

The results of this study show that SSF has the potential to be used in the develop-ment of integrated production processes for the production of innovative functional prod-ucts containing phenolic compounds in the field of dietary supplements, pharmaceuti-cals, or cosmetics.

## Figures and Tables

**Table 1:** Chemical composition of the grape pomace (GP) before (day ‘0’) and after solid-state fermentation (SSF) (5–15 days) with TV6.

Component	Untreated GP	Biologically Treated GP		
	Day ‘0’	5 Days	10 Days	15 Days
Ash [%_db_]	4.63 ± 0.03	5.28 ± 0.05	5.91 ± 0.09	6.5 ± 0.06
Crude proteins [%_db_]	14.04 ± 0.13	13.88 ± 0.14	15.75 ± 0.01	15.72 ± 0.16
Fats [%_db_]	8.69 ± 0.17	9.38 ± 0.39	9.50 ± 0.13	10.43 ± 0.39
TC [%_db_]	73.62 ± 0.17	73.98 ± 0.25	72.08 ± 0.51	73.09 ± 0.93
NDF [%_db_]	50.89 ± 0.38	58.53 ± 0.30	64.62 ± 0.44	63.82 ± 0.49
ADF [%_db_]	46.25 ± 1.17	57.88 ± 0.53	61.02 ± 0.44	60.89 ± 0.34
ADL [%_db_]	32.36 ± 0.03	39.90 ± 1.02	44.71 ± 0.58	44.15 ± 0.77
Hemicellulose [%_db_]	4.64 ± 0.78	0.65 ± 0.71	3.60 ± 0.34	2.94 ± 0.68
Cellulose [%_db_]	13.88 ± 1.19	17.98 ± 1.34	16.31 ± 0.94	16.74 ± 0.43
Sucrose [mg/g_db_]	8.83 ± 0.18	2.54 ± 0.06	1.37 ± 0.08	1.41 ± 0.08
Glucose [mg/g_db_]	1.97 ± 0.05	4.58 ± 0.18	4.72 ± 0.00	2.60 ± 0.00
Fructose [mg/g_db_]	4.56 ± 0.10	6.26 ± 0.10	0.95 ± 0.17	1.06 ± 0.11
Arabinose [mg/g_db_]	1.39 ± 0.08	3.29 ± 0.12	1.86 ± 1.46	2.06 ± 0.31
Xylose [mg/g_db_]	0.21 ± 0.04	0.46 ± 0.06	0.32 ± 0.08	0.33 ± 0.00
TOC [mg/g_db_]	47.47 ± 0.33	49.18 ± 1.41	40.26 ± 2.18	43.50 ± 3.86
TN [mg/g_db_]	1.39 ± 0.03	1.25 ± 0.07	1.57 ± 0.09	2.28 ± 0.14
TPC [mg/g_db_]	51.38 ± 1.02	22.26 ± 0.51	16.41 ± 0.58	16.84 ± 0.34
TF [mg/g_db_]	30.15 ± 0.43	13.59 ± 2.20	9.11 ± 0.40	8.08 ± 0.11
TPA [mg/g_db_]	8.79 ± 0.07	3.55 ± 0.08	2.46 ± 0.06	1.81 ± 0.04

All data are expressed as means value of replication (*n* = 3) ± SD; TC—total carbon, NDF—neutral detergent fibers, ADF—acid detergent fibers, ADL—acid detergent lignin, TOC—total organic carbon, TN—total nitrogen, TPC—total phenolic compounds, TF—total flavonoids, TPA—total proanthocyanidins, db—dry basis.

**Table 2:** Content of individual phenolic compounds in GP extract obtained before SSF (at ‘0’ day, *C*_o_) and after SSF (max. content of phenolic compounds in GP extract regardless of duration SSF and used microorganism, *C*_i, max._).

Phenolic Compounds		Microorganism	SSF (Day)	*C*_o_ (µg/g_db_)	*C*_i,max._ (µg/g_db_)
Phenolic acids (Hydroxybenzoic acids)	Gallic acid	*R. oryzae*	2	267.77 ± 11.78	568.43 ± 12.48
	Ellagic acid	*C. subvermispora*	15	34.65 ± 3.66	303.72 ± 25.89
	*p*-Hydroxybenzoic acid	*R. oryzae*	2	5.05 ± 0.22	14.37 ± 0.23
	Syringic acid	*P. eryngii*	10	86.37 ± 2.15	205.13 ± 0.01
	Vanillic acid	*R. oryzae*	2	40.54 ± 0.21	63.35 ± 1.57
	3,4-Dihydroxybenzoic acid	*H. grisea*	15	138.61 ± 9.87	699.30 ± 20.78
Phenolic acids (Hydroxycinnamic acids)	Ferulic acid	*G. lucidum*	1	4.78 ± 0.20	5.95 ± 0.21
	*o*-Coumaric acid	*T. versicolor AG613*	15	4.43 ± 0.11	33.36 ± 0.37
	*p*-Coumaric acid	*R. oryzae*	1	8.76 ± 0.70	14.63 ± 0.16
Flavan-3-ols	Epicatechin gallate	*T. versicolor AG613*	15	166.69 ± 8.42	519.13 ± 5.20
	Galocatechin gallate	*P. eryngii*	10	291.57 ± 2.35	1064.70 ± 0.25
Flavonols	Quercetin	*P. eryngii*	10	173.32 ± 16.54	662.63 ± 6.68
	Kaempferol	*P. eryngii*	10	10.22 ± 1.06	45.25 ± 0.82
Procyanidins	Procyanidin B1	*R. oryzae*	10	304.27 ± 0.37	1368.26 ± 18.19
	Procyanidin B2	*P. eryngii*	2	619.59 ± 7.90	692.75 ± 10.35
Stilbenes	Resveratrol	*P. eryngii*	5	46.07 ± 3.48	69.65 ± 0.97
	ε-Viniferin	*G. resinaceum*	4	17.52 ± 1.64	51.80 ± 0.35

Results are expressed as mean vale of replication (*n* = 3) ± SD. Student’s *t*-test was used to compare the mean values between the zero day (untreated sample) and the day of fermentation when the maximum yield of each polyphenolic compound was obtained. Statistically significant (*p* < 0.05) increase in extractability of phenolic compounds from GP after biological treatment was observed for all compounds listed in the table.

## Data Availability

The data presented in this study are available on request from the corresponding author.
